# CRISPR/Cas12a Coupled With Recombinase Polymerase Amplification for Sensitive and Specific Detection of *Aphelenchoides besseyi*

**DOI:** 10.3389/fbioe.2022.912959

**Published:** 2022-06-30

**Authors:** Anpeng Zhang, Bin Sun, Jianming Zhang, Can Cheng, Jihua Zhou, Fuan Niu, Zhongyong Luo, Luzhen Yu, Cui Yu, Yuting Dai, Kaizhen Xie, Qiyan Hu, Yue Qiu, Liming Cao, Huangwei Chu

**Affiliations:** ^1^ Institute of Crop Breeding and Cultivation, Shanghai Academy of Agricultural Sciences, Shanghai, China; ^2^ Shanghai Key Laboratory of Agricultural Genetics and Breeding, Shanghai Academy of Agricultural Sciences, Shanghai, China; ^3^ Shanghai Agricultural Science and Technology Seed Co., Ltd., Shanghai, China; ^4^ Technical Center for Animal Plant and Food Inspection and Quarantine, Shanghai Customs, Shanghai, China

**Keywords:** CRISPR/Cas12a, RPA, *Aphelenchoides besseyi*, rice, molecular identification, detection

## Abstract

*Aphelenchoides besseyi* (*A. besseyi*), a seed-borne parasitic nematode, is the causal agent of rice white tip disease (RWTD), which may result in a drastic loss of rice yield. Seed treatments are currently considered to be the most effective means of preventing the spread of RWTD. Therefore, the rapid, highly specific, and accurate detection of *A. besseyi* from rice seeds is crucial for the surveillance, prevention, and control of RWTD. Here, we describe a novel detection assay that combines recombinase polymerase amplification (RPA) and CRISPR/Cas12a to detect *A. besseyi* (termed RPA-Cas12a-Ab), with a low limit of detection (LOD) of 1 copy/μl of plasmid or 1:10^7^ diluted DNA extracted from individual nematodes*.* To improve the user-friendliness, lateral flow strip assay (LFA) was adopted to visualize the detection result. The LOD of the RPA-Cas12a-Ab LFA assay was 1,000 copies/μl plasmid or 1:10 diluted DNA extracted from individual nematodes*.* The assay developed in this study was able to identify *A. besseyi* in 45 min with high accuracy and sensitivity without cross reaction with three closely related non-*A. besseyi* species*.* Thus, RPA-Cas12a-Ab is a rapid, sensitive, and specific detection system that requires no sophisticated equipment and shows promise for on-site surveillance of *A. besseyi*.

## Introduction

*Aphelenchoides besseyi (A. besseyi)*, also known as the rice white tip nematode, has a broad host range of more than 200 plants (Cheng et al., 2013), including rice, that is, the staple food of more than half of the world’s population. *A. besseyi* is widely distributed in almost all rice-growing areas in the world, and severe infestations of *A. besseyi* may result in yield losses of up to 50% (Duncan and Moens, 2006; Cheng et al., 2013; Sánchez-Monge et al., 2015). *A. besseyi* is a seed-borne nematode, and infected seeds are considered the main source of initial inoculum for rice white tip disease (RWTD) (Devran and Göknur, 2020). It is difficult to implement a good means for thorough control of RWTD once it outbreaks (Devran and Göknur, 2020). Therefore, there is an urgent need to develop reliable and rapid detection and identification tools for *A. besseyi* (Buonicontro et al., 2018).

The conventional morphologic diagnostic method is difficult to be widely adopted due to the morphological comparability between the numerous nominal species in the *Aphelenchoides* genus (Bai et al., 2017). Therefore, molecular techniques have been the preferred methods for the identification of *A. besseyi* because of the higher sensitivity and specificity (Rybarczyk-Mydłowska et al., 2012). Currently, molecular methods for *A. besseyi* detection are mostly derived from polymerase chain reaction (PCR)-based technologies, such as loop-mediated isothermal amplification (LAMP) (Bai et al., 2017; Yang and Yu, 2019), PCR (Ibrahim et al., 1994; Devran et al., 2017; Sánchez-Monge et al., 2017), and real-time PCR (qPCR) (Rybarczyk-Mydłowska et al., 2012; Buonicontro et al., 2018; Çelik and Devran, 2019). The sensitivity of the LAMP method is similar to that of the routine PCR method, which could specifically detect target DNA levels as low as 10^3^ copies/μl (Yang and Yu, 2019), but significantly lower than that of qPCR (Buonicontro et al., 2018; Çelik and Devran, 2019). Although qPCR is the most sensitive method for detecting low concentrations of target DNA, the utilization of the qPCR method in the identification of *A. besseyi* is hampered as it is time-consuming, costly, and dependent on expensive equipment. Therefore, the development of an easy, cost-effective, and sensitive molecular method, which does not require specialized technical knowledge and auxiliary equipment, is desired (Chang et al., 2020).

Cas12a, a class II type V-A endonuclease of the CRISPR/Cas effector family, is characterized as a dual nuclease catalyzing its own CRISPR RNA (crRNA) maturation, target-site recognition, and DNA cleavage (Zetsche et al., 2015). Cas12a can detect the presence of target DNA under the guidance of crRNA. Despite mediating target-site sequence-specific DNA cleavage, Cas12a has non-specific single-stranded DNase activity (*trans*-cleavage activity) upon the formation of the Cas12a/crRNA/target DNA ternary complex (Zetsche et al., 2015; Li et al., 2018a; Chen et al., 2018). Utilizing the *trans*-cleavage activity of Cas12a, a nucleic acid detection method, named one-Hour Low-cost Multipurpose Highly Efficient System (HOLMES), was first reported in 2018 (Li et al., 2018a; Li et al., 2018b). In parallel, another similar nucleic acid detection assay, named DNA endonuclease-targeted CRISPR *trans* reporter (DETECTR), was invented by another group (Chen et al., 2018). By coupling preamplification of the target nucleic acid with PCR or isothermal amplification, the HOLMES or DETECTR assays were able to detect target DNA at a concentration as low as aM (Li et al., 2018b; Chen et al., 2018; Gootenberg et al., 2018).

The CRISPR/Cas12a-based nucleic acid detection technology has been successfully applied to detect biallelic mutants (Xiao et al., 2020), genetically modified organisms (Zhang et al., 2020; Liu et al., 2021), and various pathogens, such as the high-risk HPV16 or HPV18 virus (ZIKV) (Tsou et al., 2019), the *pseudorabies* virus (Li L. et al., 2019), the *human papillomavirus* (Gootenberg et al., 2018), the cancer-related virus (Huang et al., 2018), the white spot syndrome virus (Chaijarasphong et al., 2019), the African swine fever (Bai et al., 2019), and Magnaporthe oryzae (Zhang et al., 2020). However, to the best of our knowledge, no study has reported the use of a CRISPR/Cas12a-based assay for the molecular detection of *A. besseyi*.

**FIGURE 1:**
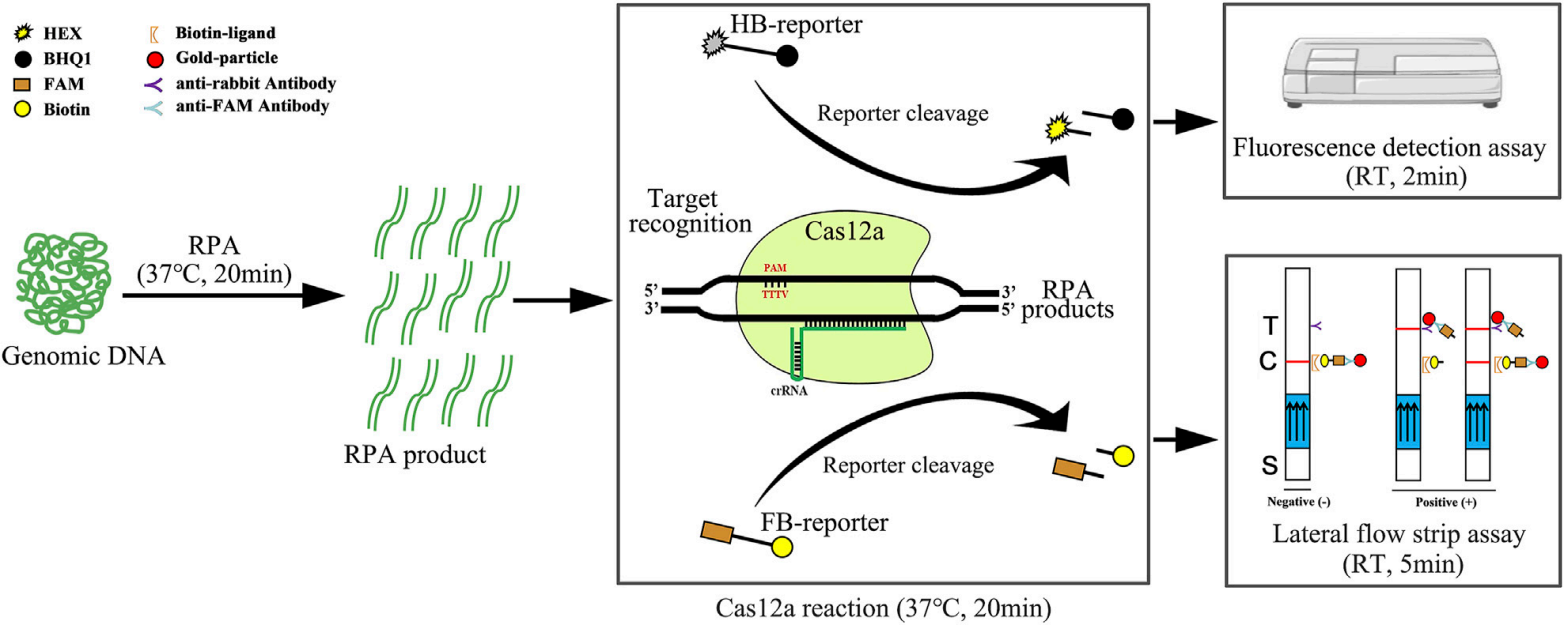
Schematic diagram of RPA-Cas12a-Ab assays using the HEX-BHQ1 reporter (HB-reporter) or FAM-Biotin reporter (FB-reporter). The HB-reporter was adopted for the fluorescence detection assay. HB-reporter was cleaved upon the activation of the *trans*-cleavage activity of Cas12a by the existence of the target DNA, and the dissociation of HEX emits fluorescence that can be conveniently detected using a fluorescence reader. Alternatively, the FB-reporter was adopted for the lateral flow strip assay (LFA). The Cas12a reaction solution is added to the sample application area (indicated by S) at the bottom of the lateral flow strip. In the absence of the target DNA, the intact FAM and biotin double-labeled probe in the Cas12a solution will be captured by the biotin ligand, and the gold-particle-conjugated anti-FAM antibody will be stuck at the control band (indicated by C). Therefore, the colored ribbon appeared on the control band only. On the contrary, in the presence of the target DNA, FB-reporter will be cleaved by the activated *trans*-cleavage activity; the dissociated FAM carrying the gold-particle-conjugated anti-FAM antibody can flow over the control band and was captured by the anti-rabbit antibody. Thus, the colored ribbon appears on the test band (indicated by T) only. In the case of low abundance of the target DNA, partial uncleaved FB-reporter would be stuck at the control band, and the colored ribbon was present on both the control band and test band. The results can be acquired in 45 min using a fluorescence reader or lateral flow strip. RT, room temperature; T, test band; C, control band; S, sample application area; arrows on the lateral flow strips indicate the flow direction of the sample.

In this study, we developed a novel detection assay that combines the CRISPR/Cas12a method with recombinase polymerase amplification (RPA) to detect *A. besseyi.* This assay has three steps, including 1) 20 min of preamplification of target DNA at 37°C, 2) 20 min of Cas12a-reaction at 37°C, and 3) readout detection results using a fluorescence analyzer or the lateral flow strip assay (LFA) within 5 min (Figure 1). Our new molecular assay (RPA-Cas12a-Ab) has the potential to detect *A. besseyi* with high accuracy, sensitivity, and specificity without the need for complicated equipment, providing a convenient and simple method that can be utilized on-site for rapid detection of *A. besseyi*.

## Materials and Methods

### Materials

The nematodes used in this study are listed in Table 1. Two rice grain samples, AB1 and AB2, infected by *A. besseyi* were collected from paddy fields in Shanghai and Nanjing, respectively. The related *Aphelenchoides* spp., *A. fragariae* (AF), and *A. subtenuis* (AS), were intercepted in Shanghai ports and saved in Shanghai Entry-Exit Inspection and Quarantine Bureau. *M. incognita* (MI) were kindly provided by Prof. Xuan Wang (Nanjing Agricultural University, Nanjing, China) (Wang et al., 2018).

**TABLE 1:** Plant-parasitic nematode species used in this study.

Sample No.	Species	Host	Origin
AB1	*A. besseyi*	*Oryza sativa*	Shanghai, China
AB2	*A. besseyi*	*Oryza sativa*	Jiangsu, China
AF	*A. fragariae*	Paeonia albiflora	Netherlands
AS	*A. Subtenuis*	Allium giganteum	Netherlands
MI	*M. incognita*	Tomato	Jiangsu, China

RPA primers, crRNA, and ssDNA reporters were synthesized by Sangon Biotech (Shanghai, China). The RPA assay kit (TwistAmp Basic kit, TABAS03KIT) was purchased from TwistDx Ltd. (TwistDx Ltd., United Kingdom). The LbCas12a (cpf1) nuclease (32108-03) was purchased from Tolo Biotech (Tolo Biotech, Anhui, China). The lateral flow strip (JY0301) was purchased from Warbio Biotech (Nanjing, China).

### Recombinase Polymerase Amplification Primer, CRISPR RNA, and ssDNAs Reporter Design

The internal transcribed spacer region (ITS) sequence of the *A. besseyi* 18S ribosomal RNA (18S rRNA) gene was retrieved from GenBank under accession number JF826519. According to the design manual of the manufacturer, RPA primers should be 30–35 bases long, and the Tm values are not the critical factor for its performance. Based on these principles, a pair of specific RPA primers with a 237 bp fragment length was designed (Table 2, Supplementary Figure S1). Eight crRNA sequences targeting the ITS of *A. besseyi* were designed based on the PAM site of Cas12a (Table 2, Supplementary Figure S1). In addition, for target detection using Cas12a *trans*-cleavage activity, the HEX-BHQ1-labeled ssDNA reporter (HB-reporter) or FAM-Biotin labeled ssDNA reporter (FB-reporter) was designed for the fluorescence assay or LFA assay, respectively (Table 2).

**TABLE 2:** RPA primer, crRNA, and ssDNA reporter sequences.

Name	Sequences (5′-3′)
RPA-F	GTT​AAT​AAG​CAC​GAA​TTA​CAG​ATA​TTA​CGA​G
RPA-R	AGT​ACT​AAC​AAC​GTA​ATC​ACA​GGC​ATA​CTC
CrRNA1	AAU​UUC​UAC​UGU​UGU​AGA​UCG​AUU​GCA​UAU​UGC​GUC​GU
CrRNA2	AAU​UUC​UAC​UGU​UGU​AGA​UCG​AUU​GCA​UAU​UGC​GUC​GUC​GGG​U
CrRNA3	AAU​UUC​UAC​UGU​UGU​AGA​UCA​UGG​AAA​GGG​AAG​CCA​AA
CrRNA4	AAU​UUC​UAC​UGU​UGU​AGA​UCA​UGG​AAA​GGG​AAG​CCA​AAU​GCA​U
CrRNA5	AAU​UUC​UAC​UGU​UGU​AGA​UGC​UAU​CGA​AUC​GUC​GAC​UC
CrRNA6	AAU​UUC​UAC​UGU​UGU​AGA​UGC​UAU​CGA​AUC​GUC​GAC​UCU​UCC​C
CrRNA7	AAU​UUC​UAC​UGU​UGU​AGA​UGU​GGC​GUA​UUC​UUC​GGA​GU
CrRNA8	AAU​UUC​UAC​UGU​UGU​AGA​UGU​GGC​GUA​UUC​UUC​GGA​GUA​UGC​C
HB-reporter	HEX-TTTTTTTTTT-BHQ1
FB-reporter	6FAM-TTTTTTTTTT-Biotin

### Construction and Purification of Reference Plasmid pUC57-18S

In order to determine the sensitivity of the RPA-Cas12a-Ab assays, a reference plasmid was constructed by cloning the 237 bp RPA fragment of 18S rRNA ITS into pUC57. The resultant plasmid, pUC57-18S, was verified by restriction enzyme digests and sequencing. The concentration of the purified pUC57-18S was determined using a NanoDrop 2000 Spectrophotometer (Thermo Scientific, United States) and calculated in copy numbers per μl and then diluted in TE buffer to a concentration of 10^10^ copies/μl.

### Nematode Extraction and DNA Isolation

The nematodes were extracted using the modified Baermann funnel method (Yang and Yu, 2019). In brief, 200 husked rice grains or other 5-g chopped plant materials infected with nematodes were wrapped with gauze and placed into a funnel filled with sterile water. After soaking for 24 h, 1,000 μl of the nematode suspension was collected from the bottom of the funnel into a 1.5 ml centrifuge tube and centrifuged for 3 min at a speed of 6,000 r/min. The supernatant was then discarded.

DNA was extracted from individual nematodes. A single nematode was transferred to a separate microtube containing 16 μl of ddH_2_O and 2 μl of 10 × PCR buffer, followed by three freeze-thaw cycles of 1 min freezing in liquid nitrogen and 2 min thawing at 95°C. 2 μl of 10 mg/ml proteinase K was added to the mixture, incubated at 65°C for 90 min, and then incubated at 95°C for 10 min to deactivate proteinase K. The supernatant was used as the DNA template. Genomic DNA (gDNA) of rice leaves was extracted from fresh leaves using the CTAB method (Borges et al., 2009).

### Recombinase Polymerase Amplification Reactions

RPA reactions were performed with a TwistAmp Basic kit (TABAS03KIT, TwistDx Ltd., United Kingdom), following the manufacturer’s instructions. In brief, the RPA reaction mixture was prepared, containing 29.5 μl of TwistAmp RPA buffer, 2.4 μl each of 10 μM RPA primer, 1 μl of the DNA template, and 12.2 μl of sterile ddH_2_O. After gentle mixing, the reaction mixture was added to the TwistAmp Basic reaction tube containing lyophilized enzyme powder. Then, 2.5 μl of 280 mM of magnesium acetate was added and mixed well, followed by incubation at 37°C for 20 min. In no template control (NTC) reaction, sterile ddH_2_O was used in place of the DNA template.

### Establishment of the RPA-Cas12a-Ab Fluorescence Assay

The Cas12a-mediated cleavage reaction was performed in a total volume of 20 μl with 2 μl 10 × Cas12a reaction buffer, 250 nM crRNA, 50 nM LbCas12a, 10 U RNase inhibitor, 250 nM HB-reporter (labeled with HEX at the 5′-end and BHQ1 at the 3′-end), and 2 μl of the RPA products. The mixture was placed in a PCR plate and then incubated at 37°C for up to 70 min in a Real-Time PCR system (Applied Biosystems, QuantStudio 6 Flex), with fluorescence signals measured every 30 s. Alternatively, the 96-well microplate was incubated at 37°C for 20 min in a fluorescence plate reader (Tecan, Infinite 200Pro) and with excitation set at 530 nm and fluorescence signals measured at 565 nm.

### RPA-Cas12a-Ab Assay With the Lateral Flow Strip Assay

The Cas12a-mediated cleavage reaction mix for LFA detection was similar as above, except that FB-reporter (labeled with 6-FAM at the 5′-end and biotin at the 3′-end) was used instead of HB-reporter (Table 2). After 20 min incubation at 37°C, the reaction products were diluted to 100 μl, after which lateral flow strips were dipped into the resultant reactions. The results were visualized after 5 min of incubation at room temperature.

## Results

### Establishing the RPA-Cas12a-Ab Fluorescence Detection Assay

**FIGURE 2:**
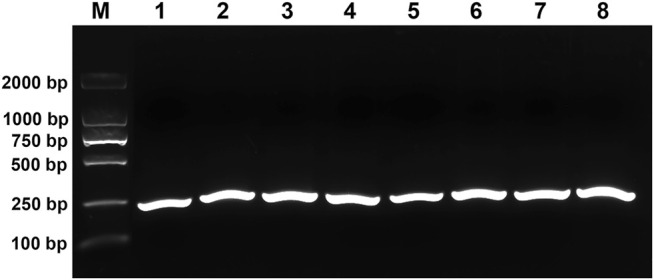
Evaluation of the performance of RPA primers. M, DL2000 DNA marker; 1–8, RPA products amplified at 25°C, 28°C, 32°C, 35°C, 37°C, 39°C, 42°C, and 45°C, respectively.

In order to evaluate the performance of RPA primers, RPA experiments were carried out using purified reference plasmid DNA as a template at eight different temperatures including 25°C, 28°C, 32°C, 35°C, 37°C, 39°C, 42°C, and 45°C. All the temperatures were able to amplify a clear and intense 237bp DNA band visualized using agarose electrophoresis (Figure 2). For the convenience of experimental operation and the minimal requirement of equipment, 37°C was chosen for subsequent RPA reactions as it is also the optimal temperature for the activity of Cas12a.

**FIGURE 3:**
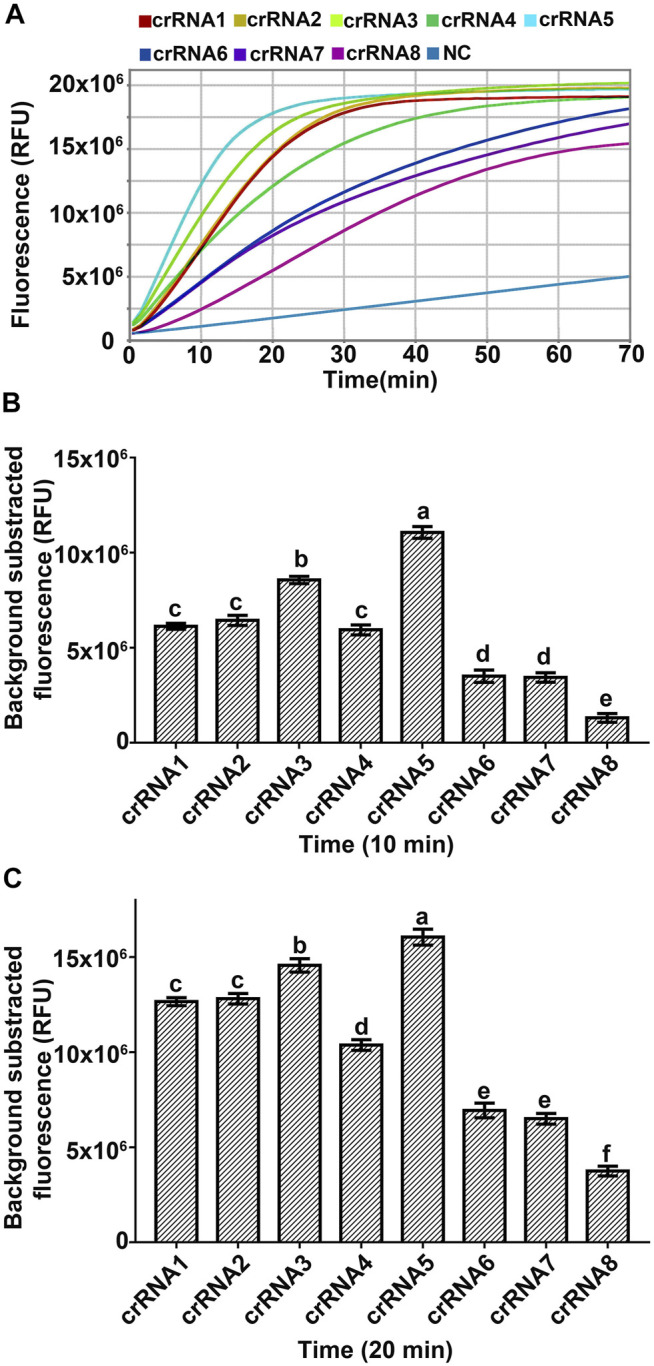
Screening an optimal crRNA for RPA-Cas12a-Ab assay with FQ-reporter. **(A)** Representative fluorescence kinetics of different crRNA using the Real-Time PCR system (Applied Biosystems, QuantStudio 6 Flex). NC, no crRNA control. **(B)** Fluorescence signals obtained at 10 min Cas12a reaction of different crRNA. **(C)** Fluorescence signals obtained at 20 min Cas12a reaction of different crRNA. The data are presented as mean ± SD (*n* = 3). crRNAs that are significantly different from each other are labeled with different lowercase letters above each bar.

crRNA is the main factor affecting the *trans*-cleavage activity of Cas12a (Creutzburg et al., 2020; Zhang et al., 2020; Liu et al., 2021; Ma et al., 2021). To obtain a better *trans*-cleavage activity of Cas12a, eight crRNAs targeting the 18S rRNA ITS of *A. besseyi* based on the PAM motif of Cas12a were designed (Table 2, Supplementary Figure S1), and the RPA-Cas12a fluorescence assay was performed to select the optimal crRNA for further experiments (Figure 1). The fluorescence signals were recorded at 30 s intervals with a QuantStudio 6 Flex Real-Time PCR System (Applied Biosystems). The fluorescence signals were detectable within 10 min for all crRNAs, among which crRNA5 generated the highest fluorescence intensity (Figures 3A–C), implying the high activity of crRNA5 for target DNA detection. The Cas12a reactions triggered by crRNA5 were completed within about 20 min, and the fluorescence kinetic curves plateaued thereafter (Figure 3A). Therefore, crRNA5 was chosen as the optimal crRNA for the RPA-Cas12a-Ab detection assay, which only takes 20 min to achieve ideal results from Cas12a reactions.

### Evaluation of the Specificity of the RPA-Cas12a-Ab Fluorescence Assay

**FIGURE 4:**
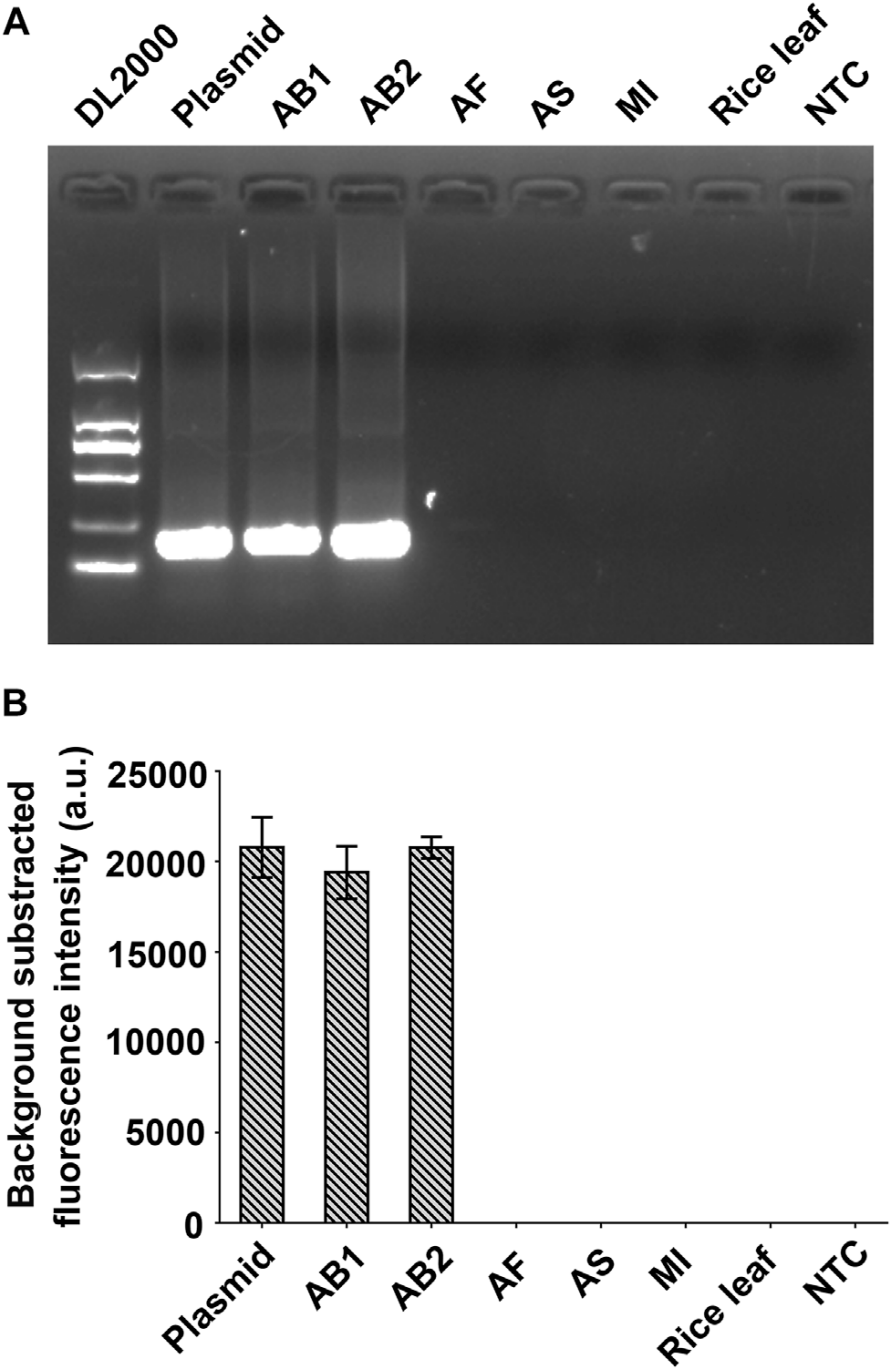
Evaluation of the specificity of the RPA-Cas12a-Ab assay. **(A)** RPA amplification of five nematodes. **(B)** Results of the RPA-Cas12a-Ab fluorescence assay. The data are presented as mean ± SD (*n* = 3). Plasmid pUC57-18S is used as the positive control; AB1, *Aphelenchoides besseyi-1*; AB2, *Aphelenchoides besseyi-2*; AS, *Aphelenchoides subtenuis*; AF, *Aphelenchoides fragariae*. MI, *Meloidogyne incognita*; NTC, no template control.

Five nematode samples, including AB1 and AB2, which were confirmed as *A. besseyi* isolates using the species-specific primers AbF5/AbR5 (Devran et al., 2017), and three non-*A. besseyi* (AF, AS, and MI) were used as templates to evaluate the specificity evaluation of the RPA-Cas12a-Ab system (Table 1, Supplementary Figure S2). RPA results show that the 237bp DNA fragment could only be amplified from *A. besseyi* samples AB1 and AB2, and not from AF, AS, and MI samples, as well as the rice gDNA and negative control (Figure 4A). Furthermore, using RPA-Cas12a-Ab fluorescence assays, AB1 and AB2 were found to have positive readings with significant fluorescence signals comparable with the plasmid positive control and did not cross-react with three non-*A. besseyi* (AF, AS, and MI) samples (Figure 4B). These results demonstrated the specificity of the RPA-Cas12a-Ab fluorescence assay.

### Evaluation of the Sensitivity of the RPA-Cas12a-Ab Fluorescence Assay

**FIGURE 5:**
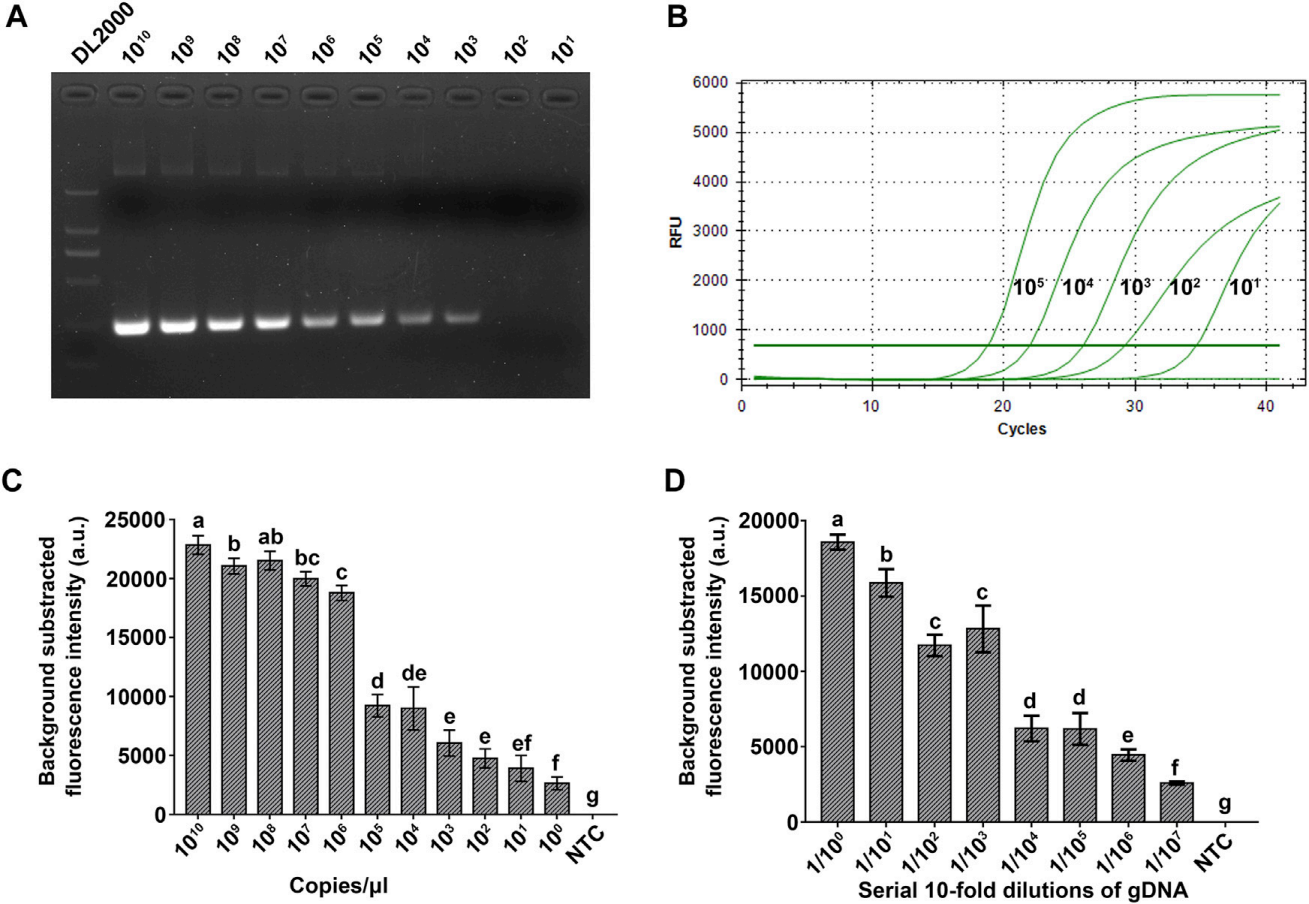
Evaluation of the sensitivity of the RPA-Cas12a-Ab assay. **(A)** Sensitivity evaluation of PCR using a serial 10-fold dilution (10^10−1^ copies/μl) of the pUC57-18S plasmid. **(B)** Sensitivity evaluation of qPCR using a serial 10-fold dilution (10^5−0^ copies/μl) of the pUC57-18S plasmid. **(C)** Sensitivity evaluation of the RPA-Cas12a-Ab fluorescence assay using a serial 10-fold dilution (10^10−1^ copies/μl) of the pUC57-18S plasmid. **(D)** Sensitivity evaluation of the RPA-Cas12a-Ab fluorescence assay using a serial 10-fold dilution (1–1/10^7^) of single *A. besseyi* nematode genomic DNA. The initial gDNA of a single *A. besseyi* is about 9.6 ng/μl. The data are presented as mean ± SD (*n* = 3). Copy numbers and serial dilution values that are significantly different from each other are labeled with different lowercase letters above each bar. NTC, no template control.

The sensitivity of the RPA-Cas12a-Ab assay was evaluated and compared with that of PCR and real-time qPCR using a serial 10-fold dilution of plasmid pUC57-18S. The limit of detection (LOD) of PCR and real-time qPCR was 10^3^ and 10^1^ copies/μl, respectively (Figures 5A,B), while the LOD of the RPA-Cas12a-Ab assay reached 1 copy/μl target DNA (Figure 5C). The RPA-Cas12a-Ab assay is thus more sensitive than PCR and qPCR in *A. besseyi* detection.

Furthermore, we used a serial 10-fold dilution of individual *A. besseyi* gDNA to assess the sensitivity of the RPA-Cas12a-Ab fluorescence assay. As shown in Figure 5D, 1:10^7^ dilution of individual *A. besseyi* gDNA was still able to generate a significant fluorescence signal as compared with the control using the RPA-Cas12a-Ab fluorescence assay (Figure 5D). The concentration of *A. besseyi* gDNA extracted from individual nematodes is about 9.6 ng/μl. Thus, the LOD of the RPA-Cas12a-Ab fluorescence assay for detection of *A. besseyi* gDNA is as low as 9.6 fg/μl.

### Establishing the RPA-Cas12a-Ab Lateral Flow Strip Assay

**FIGURE 6:**
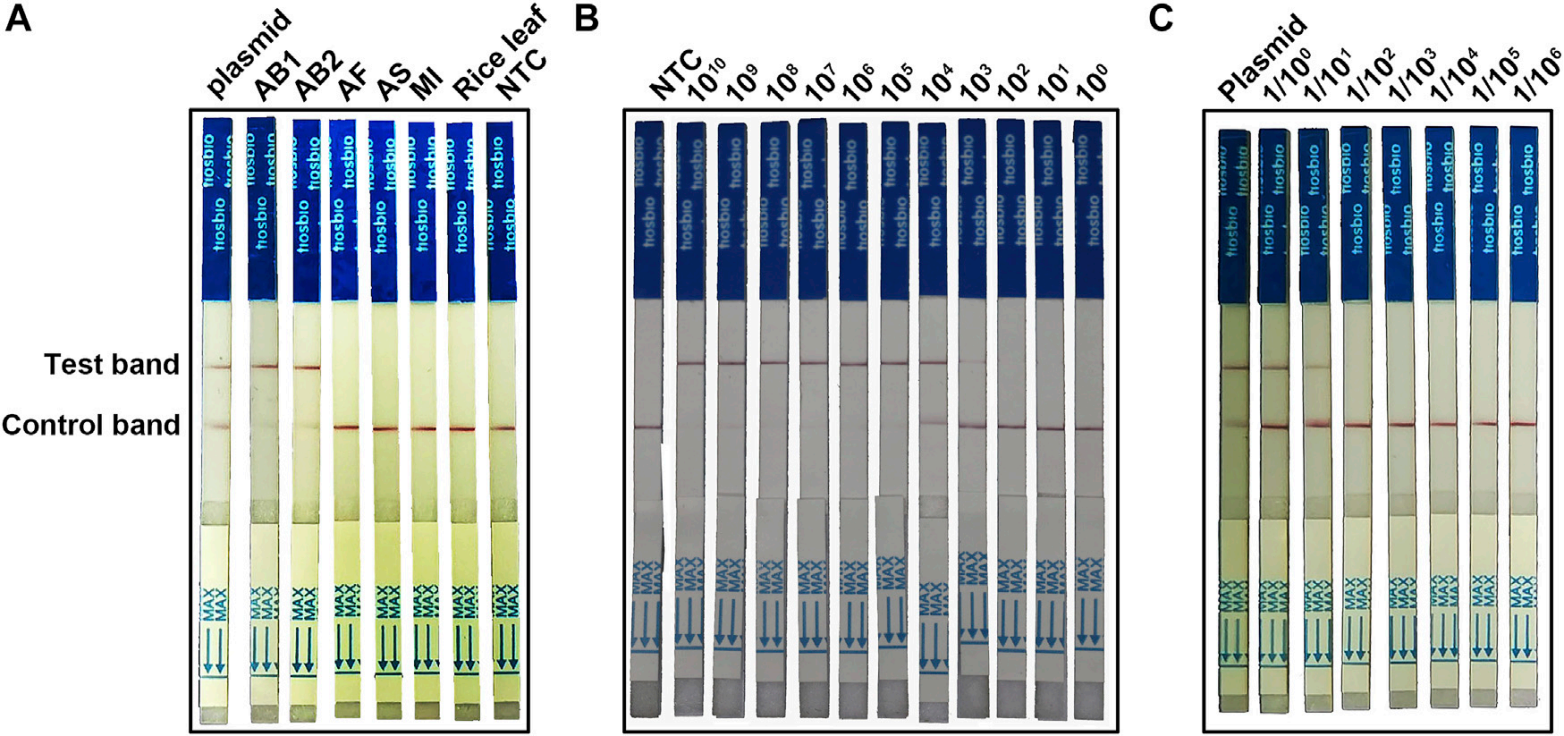
Establishing the RPA-Cas12a-Ab LFA assay. **(A)** Evaluation of the specificity of the RPA-Cas12a-Ab LFA assay. **(B)** Evaluation of the sensitivity of the RPA-Cas12a-Ab LFA assay using a serial 10-fold dilution (10^10−0^ copies/μl) of the pUC57-18S plasmid. **(C)** Sensitivity evaluation of the RPA-Cas12a-Ab LFA assay using a serial 10-fold dilution (1/10^0−6^) of single *A. besseyi* nematode gDNA. The initial gDNA of a single *A. besseyi* is about 9.6 ng/μl. Plasmid pUC57-18S is used as the positive control; AB1, *Aphelenchoides besseyi-1*; AB2, *Aphelenchoides besseyi-2*; AS, *Aphelenchoides subtenuis*; AF, *Aphelenchoides fragariae*. MI, *Meloidogyne incognita*; NTC, no template control.

For on-site detection, we combined the RPA-Cas12a with LFA technology to establish the RPA-Cas12a-Ab LFA assay (Figure 1). Five nematode samples (AB1, AB2, AF, AS, and MI, Table 1) were tested with the RPA-Cas12a-Ab LFA assay, of which *A. besseyi* samples (AB1 and AB2) showed a clear test band like that of the positive control, and no cross reactions were observed for non-*A. besseyi* samples (AF, AS, and MI). These results confirmed the specificity of the RPA-Cas12a-Ab LFA assay (Figure 6A).

A serial 10-fold dilution of reference plasmid pUC57-18S was used to evaluate the sensitivity of the RPA-Cas12a-Ab LFA assay. The LOD of the RPA-Cas12a-Ab LFA assay was 10^3^ copies/μl (Figure 6B), which was similar to that of the PCR assay (Figure 5A). We then tested the RPA-Cas12a-Ab LFA system in terms of the detection of *A. besseyi* samples. The initial gDNA extracted individual *A. besseyi* was about 9.6 ng/μl, and then the DNA was serially diluted from 1/10^0^ to 1/10^6^. The results showed that 1/10^0^ and 1/10^1^ dilutions developed a clear positive test band (Figure 6C), implying that the detection limit of the RPA-Cas12a-Ab LFA assay was 0.96 ng/μl gDNA of *A. besseyi.*

## Discussion

RWTD is a typical seed-borne disease in rice (Devran and Göknur, 2020), which is caused by the plant parasitic nematode *A. besseyi* (Jones et al., 2013). *A. besseyi* is only 0.5–1 mm in length and composed of 1000–2000 cells. It has been reported that about 92% of the rice grains had nematodes in the panicles that were infected by *A. besseyi*, and the numbers of nematodes were up to 2014 nematodes/100 grains (Lin et al., 2005). *A. besseyi* gathered inside of the glume axis of maturing grains and slowly dehydrated along with kernel drying. They become dormant and are able to survive for 8 months to 3 years during seed storage (Huang and Huang, 1974). After seed sowing, dormant *A. besseyi* rapidly reacts and embarks on a new life cycle. Therefore, accurate and rapid detection of *A. besseyi* is crucial to facilitating the quarantine of rice seeds and stopping the spread of RWTD. Due to the remarkably constrained morphology of nematodes from the same genus, molecular methods such as PCR or qPCR, have been proved helpful in detecting *A. besseyi.* However, most current PCR-based detection assays have a trade-off in performance metrics such as sensitivity and specificity and over-reliance on well-established laboratories with sophisticated facilities or well-trained operators (Li Y. et al., 2019).

In this study, we developed a new assay, the RPA-Cas12a-Ab assay, by combining RPA with CRISPR/Cas12a to detect *A. besseyi* (Figure 1). There are advantages for using this CRISPR/Cas12a-based new assay. First, unlike conventional assays, RPA-Cas12a-Ab does not rely on sophisticated equipment. Both RPA amplification and CRISPR/Cas12a reaction could be completed at body temperature, and only one fluorescence analyzer is needed to complete rapid detection. For on-site detection, we couple the RPA-Cas12a-Ab assay with LFA, allowing for readout of results without any equipment at room temperature. Second, in contrast to the time-consuming nature of conventional qPCR or PCR-based detection assays, which require 1–3 h, the newly developed RPA-Cas12a-Ab assay takes only 45 min, significantly reducing the amount of time it takes to obtain the detection results. Finally, the RPA-Cas12a-Ab fluorescence detection assay is shown to have higher detection sensitivity than conventional assays. This assay had an LOD of 1 copy/μl, which is significantly more sensitive than that of PCR and qPCR assays (Figures 5A–C). Even though the adoption of the LFA technique to readout the RPA-Cas12a-Ab detection result will increase the LOD to 10^3^ copies/μl (Figure 6B), this new assay is still robust enough to detect the presence of *A. besseyi* with high accuracy and specificity within 45 min (Figures 6A,C).

The crRNA, which plays a role in guiding Cas12a to recognize the target DNA, is critical for Cas12a-based genome editing or molecular detection. Strict selection of the target of crRNA by avoiding the homologous sites in the genome could minimize the “off-target” in genome editing (Kadam et al., 2018). Similarly, avoiding the conserved sequence in crRNA target selection is crucial for alleviating the false positive of Cas12a-based molecular detection. In this study, the ITS region sequence of the *A. besseyi* 18S rRNA gene was selected as the target sequence in RPA-Cas12a-Ab assays. The ITS region evolves much faster compared to the regions of the rRNA gene and has been widely applied as a diagnostic marker at the species level in nematodes (Powers, 2004). The specificity of the RPA-Cas12a-Ab assays was validated using three closely related non-*A. besseyi* species (Figures 4, 6A). The results showed that the assay accurately detected *A. besseyi* DNA without false-positive signals from other nematodes, confirming the specificity of RPA-Cas12a-Ab assays for *A. besseyi*.

In conclusion, we developed a new *A. besseyi* molecular detection assay that combines CRISPR/Cas12a with RPA preamplification, with readout using a fluorescence analyzer or LFA. This new assay could be completed within 45 min with no need for complicated instruments and skilled operators. The LOD of the new RPA-Cas12a-Ab assay could reach 1 copy/μl for the fluorescence detection assay and 10^3^ copies/μl for the LFA detection assay. Thus, the new RPA-Cas12a-Ab is a promising molecular method for *A. besseyi* detection with high accuracy, sensitivity, and specificity.

## Data Availability

The original contributions presented in the study are included in the article/Supplementary Material; further inquiries can be directed to the corresponding authors.
