# Supplementary material for: CRISPR/Cas12a Coupled With Recombinase Polymerase Amplification for Sensitive and Specific Detection of Aphelenchoides besseyi
Source: Front Bioeng Biotechnol. 2022 Jun 30;10:912959. doi: 10.3389/fbioe.2022.912959 (PMC9279656; doi:10.3389/fbioe.2022.912959)
Supplement: Supplementary file 1 [file Presentation1.PPTX]

## Slide 1
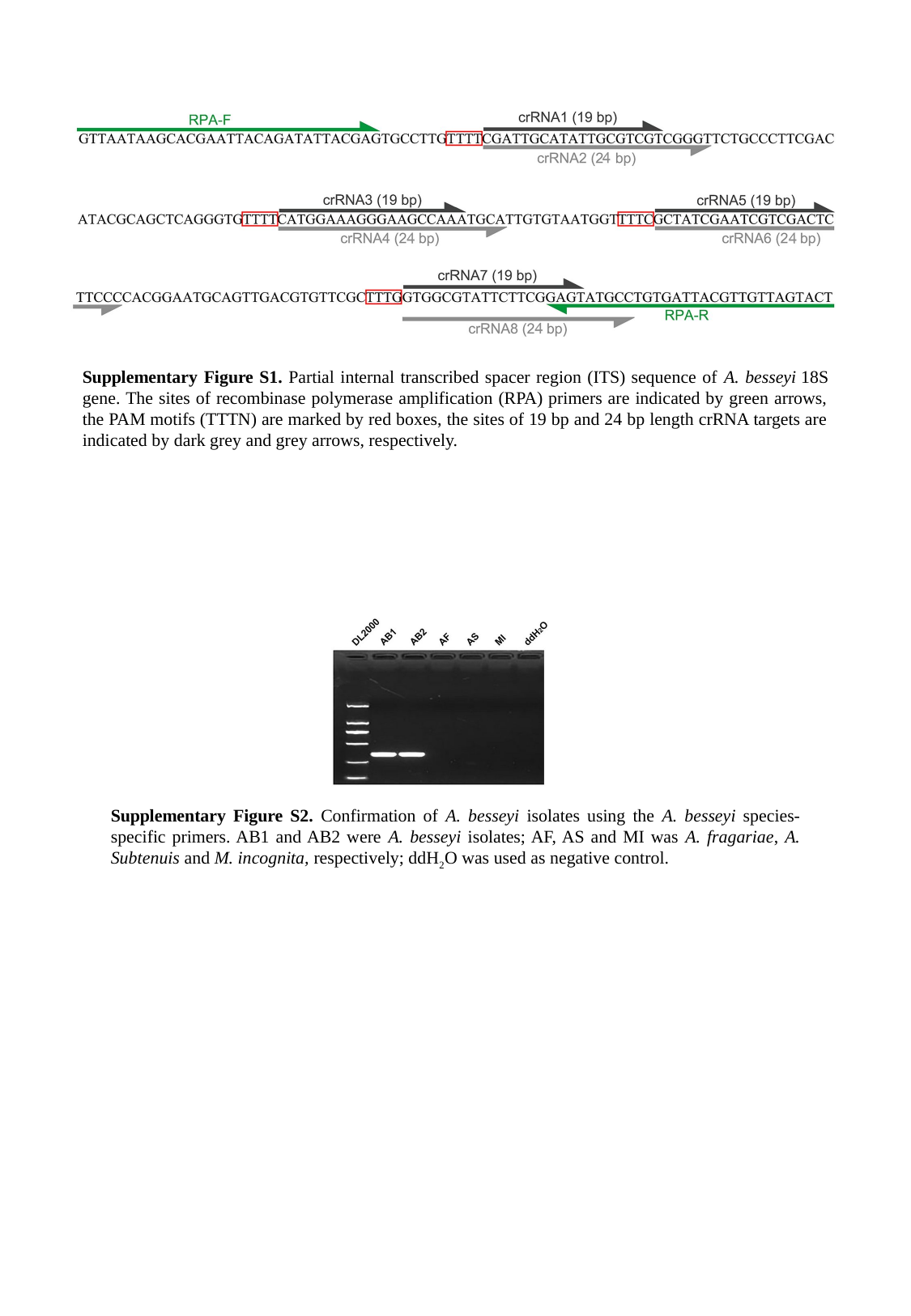

Supplementary Figure S1. Partial internal transcribed spacer region (ITS) sequence of A. besseyi 18S gene. The sites of recombinase polymerase amplification (RPA) primers are indicated by green arrows, the PAM motifs (TTTN) are marked by red boxes, the sites of 19 bp and 24 bp length crRNA targets are indicated by dark grey and grey arrows, respectively.
Supplementary Figure S2. Confirmation of A. besseyi isolates using the A. besseyi species-specific primers. AB1 and AB2 were A. besseyi isolates; AF, AS and MI was A. fragariae, A. Subtenuis and M. incognita, respectively; ddH2O was used as negative control.
